# AI-Driven Automated Detection of Pleural Plaques on Chest CT Scans in Retired Asbestos-Exposed Workers

**DOI:** 10.3390/diagnostics16162519

**Published:** 2026-08-10

**Authors:** Yannis Petitpas, Ilyes Benlala, Fabien Baldacci, Gael Dournes, Kellian Jaunas, Bénédicte Clin, Patrick Brochard, Antoine Gislard, Fleur Delva, Aude Lacourt, Christophe Paris, François Laurent, Jean Claude Pairon, Baudouin Denis de Senneville

**Affiliations:** 1 Mathematical Institute of Bordeaux (IMB), CNRS, INRIA, Bordeaux INP, UMR 5251, Université de Bordeaux, 33400 Talence, France; 2 Centre de Recherche Cardio-Thoracique de Bordeaux, INSERM U1045, Université de Bordeaux, 33000 Bordeaux, France; 3 Service d’Imagerie Médicale Radiologie Diagnostique et Thérapeutique, CHU de Bordeaux, 33000 Bordeaux, France; 4 CNRS, Bordeaux INP, LaBRI, UMR 5800, Université de Bordeaux, 33400 Talence, France; 5 Service de Médecine du Travail et de Pathologies Professionnelles, CHU de Bordeaux, 33000 Bordeaux, France; 6 Service de Santé au Travail et Pathologie Professionnelle, CHU Caen, 14000 Caen, France; clin-b@chu-caen.fr; 7 Faculté de Médecine, Université de Caen, 14000 Caen, France; 8 INSERM U1086 «ANTICIPE», 14000 Caen, France; 9 Centre de Consultations de Pathologie Professionnelle, CHU de Rouen, CEDEX, 76031 Rouen, France; 10 Epicene Team, Bordeaux Population Health Research Center, INSERM UMR 1219, Université de Bordeaux, 33000 Bordeaux, France; 11 Service Santé Travail Environnement, CHU de Bordeaux, 33000 Bordeaux, France; 12 Service de Santé au Travail et Pathologie Professionnelle, CHU Rennes, 35000 Rennes, France; 13 Institut de Recherche en Santé, Environnement et Travail, INSERM U1085, 35000 Rennes, France; 14 Equipe GEIC20, INSERM U955, 94000 Créteil, France; 15 Faculté de Santé, Université Paris-Est Créteil, 94000 Créteil, France; 16 Service de Pathologies Professionnelles et de l’Environnement, Centre Hospitalier Intercommunal, Institut Santé-Travail Paris-Est, 94000 Créteil, France; 17 Institut Interuniversitaire de Médecine du Travail de Paris-Ile de France, 94000 Créteil, France

**Keywords:** pleural plaques, presence detection, image segmentation, thoracic imaging, CT scans

## Abstract

**Background/Objective:** This study aimed to develop and validate an automated framework for detecting pleural plaques (PPs) at the patient level using a single chest CT scan. **Methods:** A database of chest CT scans with corresponding individual binary annotations for PP presence was first established through expert visual assessment, based on consensus among a board of specialized radiologists. This dataset served to train and validate a patient-level classification framework for automated PP presence detection. This framework leverages a pre-trained PP segmentation network, thus obviating the need for retraining large-scale deep learning models. This segmentation backbone is supplemented by a lightweight classification module that integrates the network’s outputs to infer the presence or absence of PPs at patient level. Furthermore, a patch-wise processing strategy was employed to optimize computational efficiency and training time while retaining critical local contextual information. **Results:** The framework was evaluated on a cohort of 1241 retired workers with documented occupational asbestos exposure, using 10-fold cross-validation (30% training, 10% validation, 60% testing). It achieved a balanced accuracy of 97.0%, sensitivity of 96.6%, and specificity of 97.4%, demonstrating performance slightly superior to that of two expert radiologists (balanced accuracy: 90.9%/91.4%, sensitivity: 88.0%/88.8%, specificity: 93.9%/94.1% for Expert1/Expert2) and substantially superior to that of a radiologist without specialized expertise in asbestos-related manifestations (balanced accuracy: 80.0%, sensitivity: 75.1%, specificity: 84.8%). **Conclusion:** The proposed framework demonstrates strong potential for automated patient-level identification of pleural plaques from a single CT scan. Our approach could be particularly useful in screening settings that involve compensation claims related to occupational diseases.

## 1. Introduction

Numerous epidemiological investigations have demonstrated a strong correlation between occupational exposure to asbestos and an increased incidence of both benign and malignant respiratory conditions. Among these, pleural plaques (PPs) represent the most frequently observed clinical manifestation [1,2,3,4,5]. Although chest radiography is widely employed as a preliminary screening method for individuals with a history of asbestos exposure, computed tomography (CT) imaging offers enhanced sensitivity and specificity for identifying pleural and parenchymal abnormalities [6,7].

In recent years, advancements in artificial intelligence have led to the development of segmentation algorithms capable of delineating PP structures in CT scans [8,9,10], generating voxel-based binary masks that facilitate the reproducible quantification of PP burden [11,12,13]. However, it is essential to distinguish between PP segmentation and patient-level detection of PP presence. Segmentation focuses on the precise localization and contouring of PP structures, whereas detection aims to determine whether PPs are present in the scan, producing a binary or probabilistic classification at the patient level. While segmentation inherently implies the presence of PPs, detection operates on a broader, clinically actionable scale. This distinction is particularly relevant in contexts where spatial localization is unnecessary or when segmentation models face challenges in identifying small, subtle, or highly variable features.

Patient-level PP detection holds significant potential as a practical imaging biomarker, especially in clinical and legal settings. For example, in France, PPs are officially recognized as a primary indicator of asbestos exposure, granting affected workers access to financial compensation and early retirement benefits. Furthermore, the prevalence of PPs has been shown to correlate with exposure-related factors [14] and is associated with an elevated risk of lung cancer [15,16]. Emerging evidence also suggests that the intraoperative identification of PPs in stage I lung cancer patients serves as an independent predictor of overall survival [17]. Notably, a substantial proportion of PPs remain undetected in preoperative CT scans, despite being confirmed intra-operatively [18], underscoring the urgent need for highly sensitive patient-level detection methodologies.

To address this gap, we propose a novel framework that explicitly targets patient-level PP detection, rather than improving PP segmentation. While our previous work introduced and validated an AI-driven pipeline for PP detection using a pre-trained segmentation network [19], it suffered from limitations: the segmentation model, trained exclusively on PP-positive cases, generated high false-positive rates due to local biases and the absence of healthy subjects in the training data. This resulted in low specificity for patient-level detection.

The key novelties of this study are as follows:

- We reformulate PP detection as a Multiple Instance Learning (MIL) problem, enabling the model to aggregate patch-level predictions into a patient-level decision, thereby improving robustness to local biases. Unlike prior studies that relied on direct thresholding of segmentation outputs, our MIL-based approach explicitly models the hierarchical relationship between local patches and global patient-level labels, improving generalization to unseen data.
- To reduce computational cost and training time while preserving local context, we introduce a patch-wise learning strategy that processes CT scans in smaller, manageable regions.
- We design a novel, lightweight machine learning classification module that bridges the gap between segmentation outputs and patient-level detection, eliminating the need to retrain large-scale deep learning models.
- We validate our framework against expert radiologists and non-specialized radiologists in a cohort of retired asbestos-exposed workers, demonstrating its superior specificity and clinical relevance compared to prior segmentation-based approaches. This study is the first to demonstrate that a patch-wise MIL framework can achieve radiologist-level performance in PP detection, paving the way for automated screening in high-throughput clinical settings.

By addressing the fundamental difference between detection and segmentation, this work advances the field beyond existing PP segmentation and preliminary detection studies, offering a scalable, interpretable, and clinically actionable solution for patient-level PP assessment.

## 2. Materials and Methods

### 2.1. General Workflow

**Figure 1:**
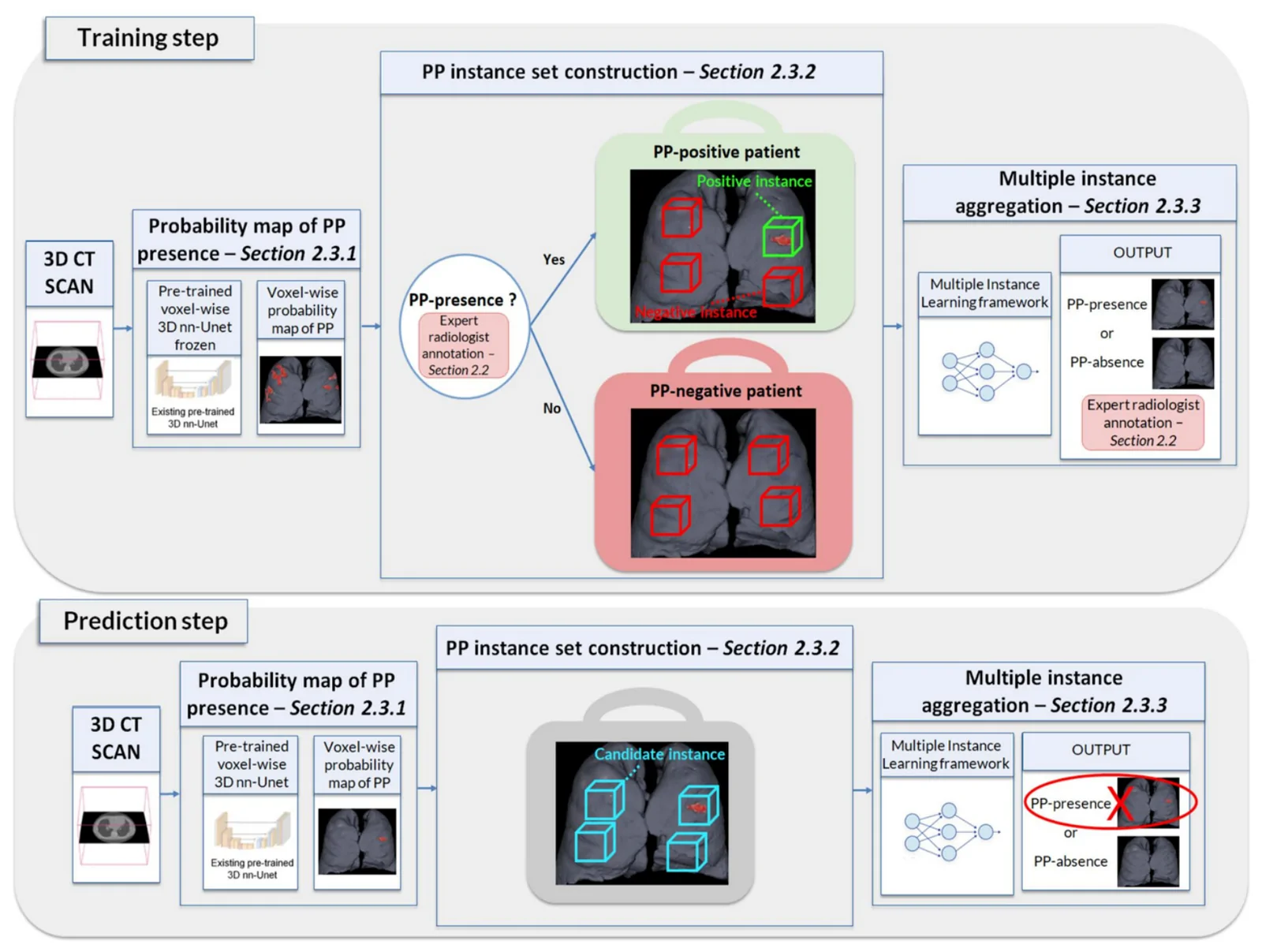
Schematic illustration of the proposed patient-level PP detection framework from chest CT scans. A voxel-wise PP probability map is first generated using a pre-trained 3D nnU-Net (Section 2.4.1). PP-candidate regions are then extracted and treated as individual instances, which are grouped into patient-level bags (Section 2.4.2). Finally, a multiple instance learning (MIL) aggregation step combines instance-level information to produce a patient-level PP presence prediction (Section 2.5).

The overall workflow of the proposed automated framework for patient-level PP detection from chest CT scans is illustrated in Figure 1. Conceptually, each individual PP region is treated as a distinct instance, while each patient is represented as a bag composed of such instances. During training, patients annotated with confirmed PP presence are labeled as positive bags, under the assumption that at least one instance corresponds to a true PP lesion, whereas patients annotated as PP-negative are treated as negative bags containing no PP-related instances. This formulation naturally enables the application of a Multiple Instance Learning (MIL) framework [20] to infer patient-level PP presence status from PP-level features.

In practice, the pipeline comprises the following sequential key steps:

1. Generation of a voxel-wise probability map of PP presence, using an existing pre-trained 3D nnU-Net applied to each chest CT scan [21]. These outputs constitute the foundation for both training and inference in the proposed classification framework.
2. Instance set construction, which refers to the extraction of PP-candidate instances and their grouping into patient-level bags.
3. Multiple instance aggregation, which combines instance-level features or predictions to produce a bag-level decision.

### 2.2. Patient Cohort

**Figure 2:**
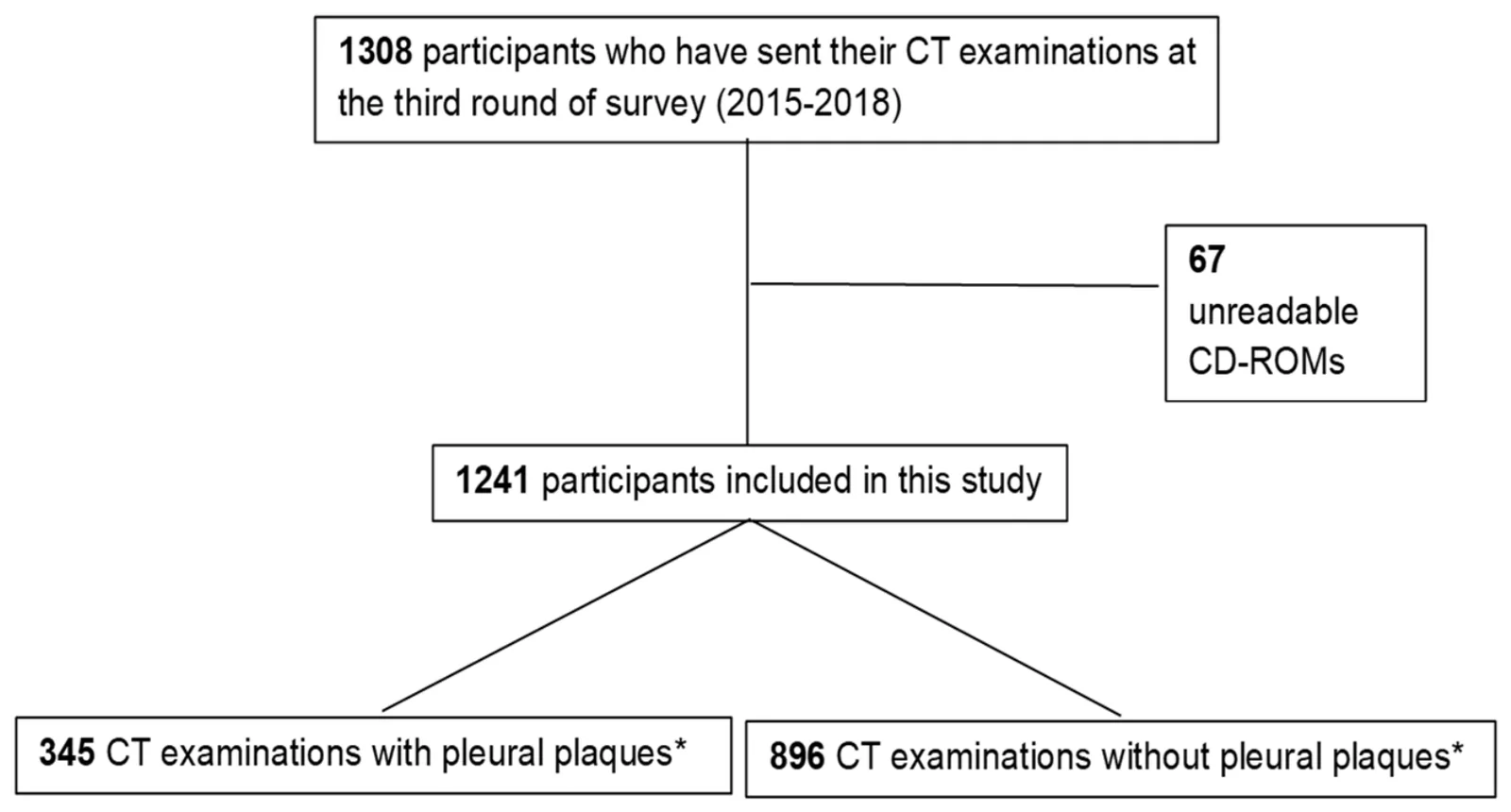
Study flow-chart of selected patients with related Computed Tomography (CT). * = with regard to the reference standard evaluation.

The proposed framework was evaluated using a multicenter cohort of retired workers [18] with documented occupational asbestos exposure. As previously described, volunteers were invited to participate using different ways and constituted the Asbestos-Related Diseases Cohort (ARDCO). They could receive a free medical check-up including chest CT-scan and pulmonary function tests [14,22,23,24]. Subjects having the CT-scan sent to the regional coordinating centers constituted the Asbestos Post-Exposure Survey (APEXS) population. A second and a third round of survey was organized 5 and 10 years after. For this analysis, we selected the most recent chest CT examinations acquired between 2015 and 2018 from the third round of screening to avoid repeated data per participant and to ensure data homogeneity. Pre-specified quality-control criteria were applied: CT examinations lacking an adequate 3D volumetric series were excluded, and only scans with a slice thickness of 1.5 mm or less and contiguous inter-slice spacing were retained. Ultimately, from the 1308 participants of the third round of screening, 1241 were included in the analysis with 67 CT data points corrupted (unreadable CD-ROMs) (see Figure 2). The hospital ethics committee approved the study (CPPRB Paris-Cochin n°1946 (2002), CPP Ile De France III n°1946/11/02-02 (2010)). All participants received information about the study and gave their written informed consent.

### 2.3. Ground Truth and Visual Evaluation

#### 2.3.1. Expert Radiologist Visual Evaluation

The presence or absence of PPs was initially determined through visual assessment of each CT scan in the full cohort of 1241 patients. Two expert radiologists independently reviewed the scans while blinded to each other’s assessments as previously described [25].

#### 2.3.2. Reference Standard Evaluation

The reference standard evaluation for the presence or absence of PPs across the entire cohort was established through consensus between the two above-mentioned expert radiologists. In cases of disagreement, a third radiologist reviewed the chest CT scans, thus resolving discrepancies as required. Using this evaluation procedure, 345 patients were classified as PP-positive and 896 as PP-negative.

#### 2.3.3. Visual Evaluation by Non-Expert Radiologists

The visual evaluation of the radiologists without specialized expertise in asbestos-related manifestations including PPs, available in the initial reports of scans, were collected as part of the ARDCO study and used in our analysis.

### 2.4. Image Pre-Processing

#### 2.4.1. Generation of Voxel-Wise Probability Maps of PP Presence

To obtain spatially resolved estimates of PP presence throughout the lung volume, a pre-trained 3D nnU-Net model was applied to each chest CT scan [21]. All CT volumes were initially resampled to an isotropic voxel resolution of 1 × 1 × 1 mm^3^ and processed using the standard preprocessing pipeline embedded in the pre-trained nnU-Net framework [26]. No explicit denoising or additional image-filtering techniques were applied prior to inference. This model, previously optimized for PP segmentation in prior studies, generated voxel-wise probability maps that served as the foundation for both the training and inference phases of the proposed framework. Unlike conventional segmentation approaches, which produce binary masks, the nnU-Net was configured to output continuous probability values by omitting the final activation layer. This modification preserved the model’s confidence at each voxel, enabling each voxel value to represent the estimated likelihood of PP presence.

#### 2.4.2. PP Instance Set Construction for Patient-Level Prediction

*Patch-wise representation of PP-features:* To mitigate computational costs and reduce training time while preserving essential local contextual information, a patch-wise data representation strategy was adopted. To this end, connected components were first extracted from the binary PP segmentation map obtained by thresholding the voxel-wise PP probability map at 0.5. Only the top *N* connected components with the highest predicted PP presence probabilities were retained for further analysis. To assess the influence of this candidate-selection parameter, four values were evaluated: N = 16, 32, 64, and 100. Within each selected component, the voxel exhibiting the maximum PP probability was identified and used as the center for extracting a standardized 3D patch. Cubic patches of multiple sizes (9 × 9 × 9, 17 × 17 × 17, and 33 × 33 × 33 voxels) were systematically evaluated to assess the impact of spatial context on model performance.

*Patch-filling strategy:* To assess the relative contribution of probability information versus imaging features, three patch-filling strategies were evaluated: Single-Channel probability patches (denoted as “SC-proba”), Single-Channel CT intensity patches (denoted as “SC-CT”), and Dual-Channel probability/CT patches (denoted as “DC-proba/CT”).

- “SC-proba” strategy: Single-Channel patches containing PP probability values derived from nnU-Net (this strategy is defined here and used as the default patch-filling approach throughout the remainder of the manuscript).
- “SC-CT” strategy: Single-Channel patches containing normalized CT intensity values.
- “DC-proba/CT” strategy: Dual-Channel patches combining probability maps with normalized CT patches.

For the DC-proba/CT strategy, CT intensity values were normalized to the range (0, 1) at the patch level to avoid scale discrepancies with the PP probability maps.

*Patient-level bag construction:* Each patient was represented as a bag composed of multiple PP instance-level patches. During training, patients annotated with confirmed PP presence were labeled as positive bags, under the assumption that at least one instance corresponds to a true PP lesion, whereas patients annotated as PP-negative were treated as negative bags containing no PP-related instances.

### 2.5. Proposed Network for PP Detection at the Patient Level

#### 2.5.1. Model Architecture

To classify patient-level PP status, we employed an MIL framework, treating each patient as a bag of instances (patches). A max-pooling aggregation strategy was employed to propagate the most predictive PP-related signal from the instance level to the bag level. We independently evaluated three deep learning-based classification architectures: ResNet-18, ResNet-50 and ResNext [27]. Supplementary Figure S1 presents a schematic representation of the MIL architecture employed in our workflow. The figure illustrates the integration of a ResNet-18 backbone to extract instance-level features, which are subsequently aggregated to generate a patient-level prediction of PP presence.

#### 2.5.2. Model Training

The model was trained from scratch using the Adam optimizer, with a batch size of two patches per iteration. The binary cross-entropy (BCE) loss was employed, and class labels were encoded as 0 for PP-negative patients and 1 for PP-positive patients. Training was conducted for a maximum of 100 epochs, employing an adaptive learning rate reduction strategy: the learning rate was halved whenever the validation performance failed to improve for five consecutive epochs. Random flipping of extracted patches was used as data augmentation and was applied only to the training subset and was not applied to validation or test data. To mitigate the effect of class imbalance, training batches were balanced between PP-positive and PP-negative samples. To minimize overfitting, early stopping was implemented based on the validation balanced accuracy, and the model achieving the highest validation balanced accuracy was retained for subsequent evaluation.

#### 2.5.3. Inference and Score Aggregation

Model checkpoint selection was based on validation balanced accuracy. The inference threshold was optimized to favor validation sensitivity, in order to reflect the clinical diagnostic context, in which false-negative predictions may lead to missed pathological findings and delayed patient management, whereas false positives can be further addressed through complementary diagnostic assessments.

#### 2.5.4. Hyper-Parameter Tuning on a Held-Out Exploration Subset

Hyper-parameter tuning was performed on the exploration cohort to optimize model performance, with a focus on maximizing sensitivity while maintaining balanced accuracy.

### 2.6. Alternative Approaches

To benchmark the performance of our proposed automated method, we conducted a comparative analysis against the two aforementioned expert radiologists, as well as a non-expert reader. Furthermore, we evaluated a standalone segmentation-threshold baseline, a straightforward machine learning classification approach, adapted from our preliminary work on PP presence detection [19]. In this alternative method, a patient was classified as PP-positive if at least one voxel within a patch exceeded a tunable inference probability threshold. The threshold was optimized on the training set to maximize balanced accuracy between predicted PP presence/absence and the reference-standard evaluations, employing Brent’s optimization method.

### 2.7. Experimental Evaluation Protocol

#### 2.7.1. Training and Inference via Cross-Validation

The dataset was partitioned into training, validation, and testing subsets, stratified by the presence of PPs to maintain a distribution of 30% training, 10% validation, and 60% testing. Performance metrics were computed across 10 Monte Carlo cross-validation trials with random resampling to ensure robustness and generalizability of the results. Hyper-parameter tuning, model checkpoint selection and inference-threshold optimization were conducted solely on the training and validation subsets.

#### 2.7.2. Statistical Analysis

The following performance metrics were computed: balanced accuracy, sensitivity, specificity, positive predictive value (PPV), and negative predictive value (NPV). For each metric, the mean, standard deviation, and 95% confidence interval (95% CI) were reported to provide a comprehensive assessment of model performance.

To evaluate the influence of patch selection, classification models, and patch-filling strategies, pairwise performance differences were assessed for statistical significance using a paired Wilcoxon signed-rank test. A *p*-value < 0.05 was considered statistically significant.

#### 2.7.3. Hardware Specifications

All training computations were performed on a high-performance workstation equipped with an Intel(R) Xeon(R) Gold 6248R CPU and an NVIDIA RTX A6000 GPU featuring 48 GB of VRAM. For inference benchmarking of the complete processing pipeline, experiments were conducted on the same workstation. To further assess the feasibility of deployment in realistic clinical scenarios, such as during a general practitioner’s consultation, we also evaluated the pipeline on a consumer-grade laptop equipped with an Intel Core i5-1335U processor (without dedicated GPU) and 32 GB of RAM.

#### 2.7.4. Implementation Details

The entire implementation was developed in Python 3.9. The SimpleITK library [28] was employed for handling CT images and managing probability maps, including connected component extraction. Visualization of intermediate and final results was performed using the Matplotlib library 3.9.0 [29]. The MONAI [30] and PyTorch [31] frameworks were used to implement and train the ResNet-18 and ResNet-50 architectures. Evaluation metrics were computed with the scikit-learn library [32].

## 3. Results

**Figure 3:**
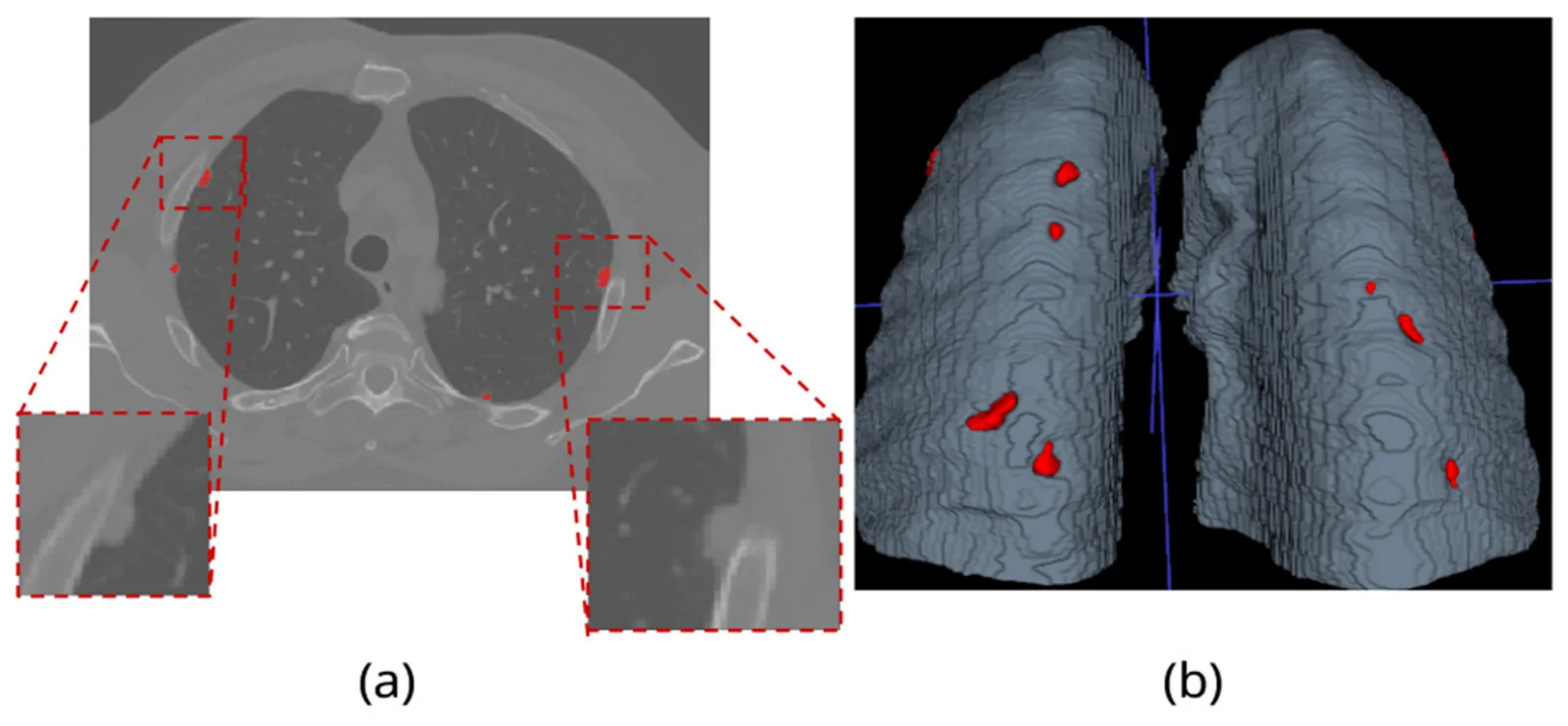
Representative CT chest scan from a retired asbestos-exposed worker. (**a**) Transverse cross-section of the lung CT image with PP segmentations overlaid in red, obtained using a pre-trained PP segmentation network. The inset provides a magnified view of the segmented PPs for improved visualization. (**b**) 3D rendering of the segmented PPs (in red), illustrating their distribution on the outer surface of both lungs (the lungs volume rendering in grey).

Figure 3 illustrates a representative CT scan from a retired asbestos-exposed worker. Panel (a) shows a transverse cross-section of the lung with PP segmentation overlaid in red, obtained using the existing pre-trained PP segmentation network (Section 2.3.1). The inset provides a magnified view, where non-calcified PPs can be observed at the lung periphery. Their small size and attenuation values, which are close to those of adjacent muscle tissue, highlight the inherent difficulty of accurate PP segmentation. Panel (b) presents a 3D rendering of the segmented PPs (red), illustrating their distribution on the outer surface of both lungs (grey).

**Figure 4:**
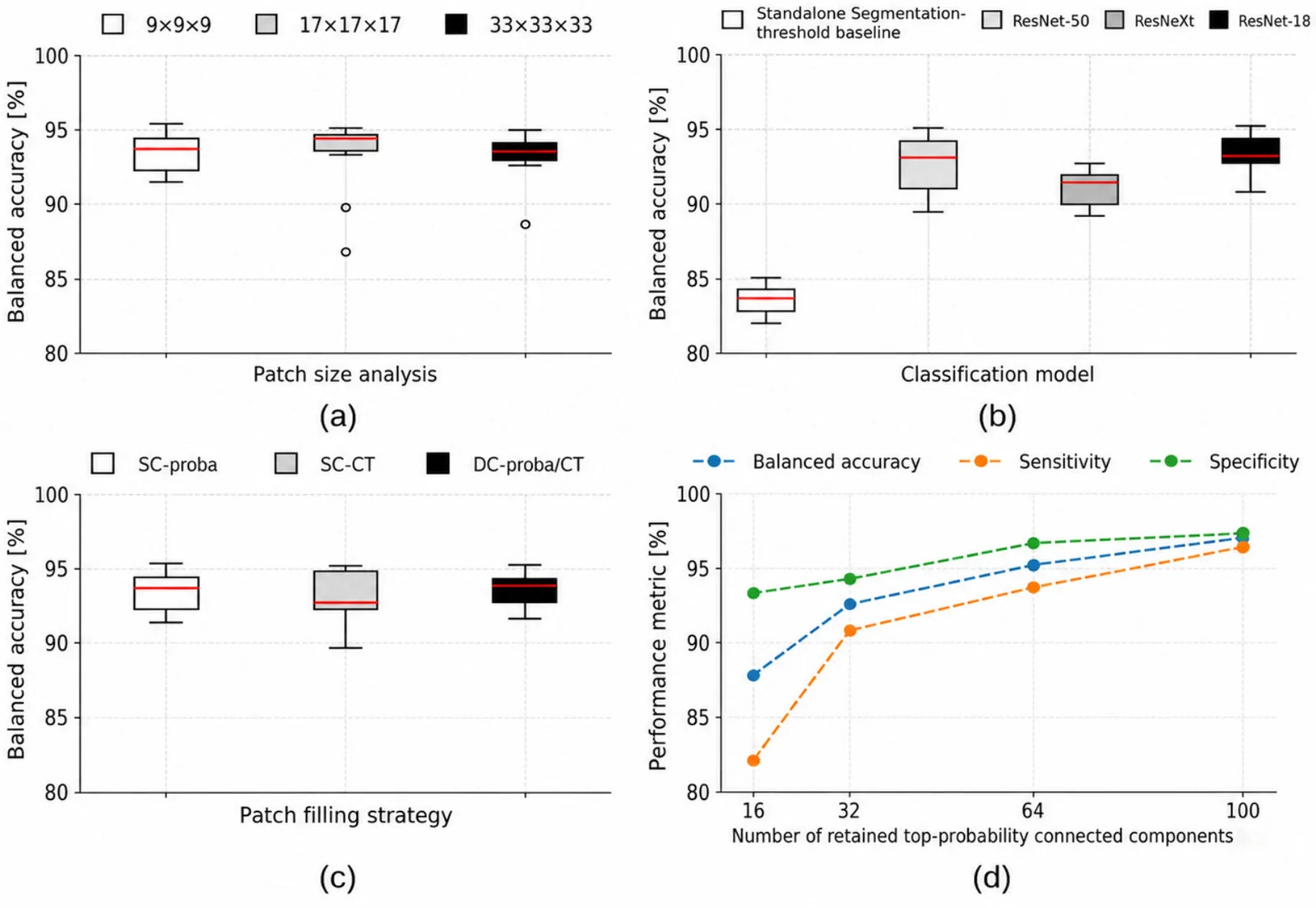
Quantitative evaluation of the proposed framework for patient-level PP presence detection. (**a**) Influence of patch size using a ResNet-18 classifier with patches filled using the SC-proba strategy, with the top N = 32 connected components retained. (**b**) Comparison of classification algorithms used to link voxel-wise segmentation outputs to patient-level PP detection, including the standalone segmentation-threshold baseline, ResNet-18, ResNet-50, and ResNeXt classifiers using 9 × 9 × 9 patches filled with the SC-proba strategy, with the top N = 32 connected components retained. (**c**) Influence of the patch-filling strategy, comparing SC-proba, SC-CT, and DC-proba/CT, with the top N = 32 connected components retained. (**d**) Influence of the number of retained top-probability connected components N on balanced accuracy, sensitivity, and specificity. In panels (**a**–**c**), the red line denotes the median, boxes represent the first and third quartiles, and dots indicate outliers.

A quantitative evaluation of the proposed framework is presented in Figure 4. Panel (a) examines the effect of patch size using a ResNet-18 classifier with the SC-proba input. No statistically significant differences were observed across patch sizes (*p* > 0.05 for all paired comparisons). Panel (b) compares the aggregation methods used to combine patch-level predictions for patient-level PP detection. Deep learning-based approaches (ResNet-18 and ResNet-50) outperformed alternative classifiers, with no statistically significant difference between the two ResNet architectures. It is noteworthy that our investigation also extended to more recent deep learning architectures. However, ConvNeXt [33], which is optimized for large-scale images with rich spatial context, demonstrated incompatibility with the small patch sizes required for this study. Furthermore, ResNeXt [27] did not provide a consistent advantage compared to the 3D ResNet architecture, as quantitatively illustrated in Figure 4, panel (b). Supplementary Figure S2 displays the training and validation loss functions, along with the balanced accuracy curves. These curves indicate overfitting, where the model memorizes the training data but fails to generalize to unseen validation data. Despite being the smallest official ResNet variant, the ResNet-18 architecture remains overly complex (i.e., parameter-rich) for our dataset. Optimization tests for this network only marginally mitigated this effect but did not resolve it. To address this, we implemented early stopping as a regularization strategy. Early stopping interrupts the training process when validation performance reaches a plateau or starts to decline, thereby mitigating overfitting to the training data and enhancing the model’s generalization capability.

Panel (c) evaluates the impact of the patch-filling strategy, showing that the SC-proba, SC-CT, and DC-proba/CT inputs resulted in comparable performance, with no significant differences between configurations (*p* > 0.05 for all paired comparisons). The highest performance was obtained using a ResNet-18 architecture with 9 × 9 × 9 patches filled with the SC-proba strategy, yielding a balanced accuracy of 97.0% (95% CI: 96.2–97.7), a sensitivity of 96.6%, and a specificity of 97.4% (the confusion matrix on the test dataset is provided in Supplementary Figure S3). The workflow retains only the top N connected components from the PP segmentation, ranked by maximal probability. The potential risk of omitting true PP regions due to this top-N selection strategy is addressed in Panel (d), which illustrates that sensitivity and specificity metrics improve monotonically with increasing N, converging toward a plateau at approximately 97% at N = 100, suggesting that true pathological events are effectively captured within this range.

Table 1 summarizes the performance of the proposed framework for patient-level PP detection and compares it with that of one non-expert radiologist and two expert radiologists. All comparisons were performed against the reference-standard evaluation on the test subsets of the 10 repeated Monte Carlo train/validation/test splits. The proposed framework achieved a balanced accuracy of 97.0%, surpassing those of the two expert radiologists (90.9% for Expert 1 and 91.4% for Expert 2), while also outperforming the non-expert radiologist, who achieved a balanced accuracy of 80.0%.

In terms of computational cost, the generation of the probability map for PP presence using the existing pre-trained network (Section 2.3.1) accounted for the majority of the total processing time. Computation was moderate on our workstation platform (7 min 52 s). Once the probability map of PP presence was obtained, the proposed lightweight classification module was able to predict the binary presence or absence of PPs at the patient level within a reasonable time frame on both systems: 12 s on the workstation and 38 s on the consumer-grade laptop.

## 4. Discussion

In this study, we proposed and validated a comprehensive automated deep learning framework for patient-level detection of PPs from a single CT scan. To the best of our knowledge, previous studies on automated PP analysis have primarily focused on lesion detection or segmentation rather than patient-level classification. Early computer-aided detection approaches aimed to localize PPs on chest CT scans using handcrafted features and knowledge-driven methods [34]. More recently, deep learning approaches have been developed to detect PPs at the lesion level [8] or to automatically segment and quantify plaque burden in asbestos-exposed individuals [11]. In contrast, the present work addresses a distinct task by formulating PP assessment as a binary patient-level classification problem, where the objective is to determine whether a chest CT examination contains at least one PP. Rather than localizing or segmenting individual lesions, our approach directly predicts the presence or absence of PPs at the patient level. This formulation is particularly relevant for large-scale screening and triage applications, where a rapid and reliable identification of patients with PPs may be sufficient to guide subsequent clinical review.

To achieve this, we integrated an existing pre-trained PP segmentation network into a complete pipeline specifically designed to determine the presence or absence of PPs at the patient level. It should be emphasized that PP segmentation in CT scans remains inherently challenging due to the small size, multiplicity, and low contrast of PPs relative to the thoracic wall, especially thoracic muscles. This difficulty is further exacerbated by the presence of other fine structures in the lung, which can introduce local bias and limit PP detection in certain individuals. Moreover, the existing pre-trained network was trained exclusively on patients with PPs and did not include healthy subjects, resulting in a potentially highly unbalanced dataset for local PP segmentation [26]. Consequently, when applied directly to patient-level PP detection, the segmentation model generated a large number of false-positive predictions, resulting in only moderate specificity, even when an optimized inference threshold was applied, as shown in Table 1. To address these limitations, we propose a deep learning classifier that bridges local PP segmentation outputs and patient-level PP detection. This approach enables the pre-trained PP segmentation network to be seamlessly integrated into a fully automated PP detection framework without the need to retrain a large segmentation model.

It should be emphasized that incorporating anatomical CT information in addition to the PP probability map did not result in a statistically significant improvement in PP presence classification performance in our experiments (*p* > 0.05). This behavior was consistent across all evaluated patch sizes, with no clear trend indicating an advantage from multi-channel input representations. These findings suggest that the proposed framework is relatively insensitive to several key hyper-parameters, which is a desirable property in practical applications. In particular, the limited impact of input representation complexity indicates that reliable patient-level PP detection can be achieved using lightweight architectures and reduced data representations. This robustness not only simplifies model design and training but also facilitates deployment in resource-constrained clinical settings, while reducing the risk of over-fitting and improving reproducibility across datasets.

Existing results from the standalone segmentation-threshold baseline, as reported in the literature [19], serve as a reference for comparison. The baseline approach, which relies on direct thresholding of segmentation probability maps, demonstrated inferior performance compared to the proposed MIL-based framework. This outcome underscores the limitation of segmentation or local detection models in translating to reliable patient-level classification, as direct thresholding proves insufficient for robust detection at the patient level. The proposed MIL classification module addresses this limitation by introducing an additional layer of false-positive reduction and aggregation. It learns to identify which candidate regions are most informative for determining patient-level PP status.

In the context of automated PP detection, the present study achieves performance comparable to that of expert readers and significantly outperforms non-specialized radiologists. From a clinical perspective, the main contribution of the proposed framework is to provide automated patient-level PP detection from a single chest CT scan. This could support post-occupational surveillance and compensation-related workflows in asbestos-exposed workers by identifying CT examinations with a high likelihood of PP presence and prioritizing them for expert radiological review. The framework is not intended to replace expert assessment, but rather to act as an assistance or triage tool, particularly in settings involving large numbers of CT examinations or non-specialized readers. It is noteworthy that our framework demonstrates superior sensitivity compared to expert radiologists while maintaining high specificity, particularly when benchmarked against non-expert radiologists. This distinction holds significant clinical relevance, as occupational physicians face the challenge of efficiently triaging the growing volume of chest CT scans. By accurately identifying cases with a high likelihood of pleural plaque presence, our approach enables targeted referral for expert opinion, thereby optimizing resource allocation and diagnostic workflows.

One notable aspect of our methodology is the unconventional data partitioning strategy, which allocated 30% of the dataset to training, 10% to validation, and 60% to testing. While traditional deep learning paradigms often favor larger training sets to optimize model convergence, our approach was justified by the substantial size of our cohort (1241 patients), ensuring that even the 30% training subset (372 patients) retained sufficient statistical power for effective learning. Furthermore, the emphasis on a larger test set was intentional, prioritizing rigorous evaluation of the model’s generalization capability. This strategy was further validated through multiple randomized trials, which demonstrated consistent performance metrics across distinct data splits, thereby mitigating concerns about over-fitting or partitioning bias. Thus, our partitioning reflects a deliberate trade-off between training robustness and the need for a comprehensive assessment of real-world applicability.

With regard to the practical deployment of this framework in realistic clinical environments, it is important to highlight that the inference time of the pre-trained nnU-Net represents the predominant contributor to the overall computational latency. However, once the probability map for PP presence was generated, the proposed supplementary module efficiently predicted the binary outcome (presence or absence of PP) at the patient level within a clinically acceptable timeframe (less than one minute). These results suggest that the computationally intensive step of generating the PP probability map should be offloaded to local or cloud-based solutions, which are commonly integrated into commercially available medical imaging platforms. This approach would ensure both scalability and practicality in real-world clinical workflows.

Several limitations should be acknowledged. First, while max-pooling provides a straightforward approach for instance aggregation, its sensitivity to outliers or false positives is a known limitation. Future work could explore advanced aggregation methods, including attention-based MIL, to mitigate these issues and improve the reliability of patient-level predictions. Second, it is important to acknowledge that the generalizability of our findings may be constrained by the specific characteristics of our study population, which consisted of retired workers with occupational exposure to asbestos, and this may limit the generalizability of our results to populations undergoing CT for other indications, particularly incidental lesion detection in routine clinical practice. Further validation in broader clinical populations will therefore be necessary to confirm the applicability of the model across different settings.

## 5. Conclusions

In this study, we developed and validated an automated deep learning framework for patient-level detection of PPs using CT scans. By integrating a pre-trained PP segmentation network with a lightweight classification module, the proposed approach effectively links local segmentation outputs to patient-level predictions without the need for retraining a large model.

The framework achieved expert-level performance for PP detection in a cohort of retired asbestos-exposed workers, outperforming experienced radiologists and clearly surpassing a non-expert reader.

Clinically, this approach could support post-occupational surveillance and compensation-related workflows by assisting patient-level PP identification and prioritizing cases for expert review. External validation in independent and more heterogeneous cohorts remains necessary before broader clinical deployment.

## Figures and Tables

**Table 1:** Comparison of performance between expert radiologists, non-expert readers, and the proposed automated MIL approach (ResNet-18 classifier using 9 × 9 × 9 patches filled with the “SC-proba” strategy; top N = 100 connected components in PP segmentation retained) in a cohort of retired asbestos-exposed workers. Performance was evaluated against reference-standard evaluations. Balanced accuracy, sensitivity, specificity, PPV, and NPV are reported at the patient level. Results are expressed as mean ± standard deviation, with 95% confidence intervals shown in brackets obtained using cross-validation.

Patient-Level PP Detection Method	Balanced Accuracy [%]	Sensitivity [%]	Specificity [%]	PPV [%]	NPV [%]
Non-expert radiologist	80.0 ± 0.9 CI [78.7; 81.3]	75.1 ± 1.9 CI [72.8; 77.4]	84.8 ± 0.9 CI [83.5; 86.1]	68.1 ± 3.6 CI [64.1; 72.1]	88.9 ± 1.4 CI [87.1; 90.7]
Expert 1	90.9 ± 0.8 CI [90.3; 91.5]	88.0 ± 1.2 CI [87.1; 88.8]	93.9 ± 0.7 CI [93.4; 94.4]	84.7 ± 1.6 CI [83.6; 85.8]	95.3 ± 0.5 CI [95.0; 95.6]
Expert 2	91.4 ± 0.8 CI [90.8; 92.0]	88.8 ± 1.5 CI [87.7; 89.8]	94.1 ± 0.6 CI [93.7; 94.5]	85.2 ± 1.3 CI [84.3; 86.1]	95.6 ± 0.6 CI [95.2; 96.0]
Standalone segmentation-threshold baseline	83.6 ± 0.9 CI [82.9; 84.3]	82.7 ± 3.0 CI [80.6; 84.9]	84.5 ± 2.1 CI [83.0; 86.0]	67.3 ± 2.4 CI [65.6; 69.0]	92.8 ± 1.0 CI [92.0; 93.5]
Proposed MIL framework	97.0 ± 1.1 CI [96.2; 97.7]	96.6 ± 2.7 CI [94.7; 98.5]	97.4 ± 0.9 CI [96.7; 98.0]	93.4 ± 1.9 CI [92.0; 94.8]	98.7 ± 1.0 CI [97.9; 99.4]

## Data Availability

Data are available under reasonable request.

## Supplementary Materials

The following supporting information can be downloaded at https://www.mdpi.com/article/10.3390/diagnostics16162519/s1, Figure S1: Schematic representation of the MIL architecture employed in our workflow. The ResNet-18 backbone processes instance-level features to generate a patient-level prediction of PP presence; Figure S2. Training and validation loss and balanced accuracy curves over 100 training epochs for the ResNet-18 classifier using 9 × 9 × 9 patches generated with the SC-proba strategy (top N = 100 connected components retained). Solid lines represent the mean performance across 10 independent training runs, and the shaded regions indicate the corresponding standard deviation. (a) Training (blue), validation (red), and validation with early stopping (green) loss curves. (b) Training (blue), validation (red), and validation with early stopping (green) balanced accuracy curves. Early stopping was applied based on validation balanced accuracy to mitigate overfitting, and the model with the highest validation balanced accuracy was retained for subsequent evaluation. The green curves illustrate the performance of the selected models after early stopping, confirming its effectiveness in stabilizing validation loss and maintaining high balanced accuracy; Figure S3. Confusion matrix for patient-level PP detection on the test dataset using the proposed MIL framework. Values represent the mean row-normalized percentages of true negatives (TN), false positives (FP), false negatives (FN), and true positives (TP) across 10 Monte Carlo cross-validation runs.

## Abbreviations

The following abbreviations are used in this manuscript:

PP	Pleural plaques
CT	Computed tomography
MIL	multiple instance learning
SC-proba	single-channel patches containing PP probability
SC-CT	single-channel patches containing normalized CT
DC-proba/CT	strategy: dual-channel patches combining probability maps with normalized CT patches
