# Supplementary material for: AI-Driven Automated Detection of Pleural Plaques on Chest CT Scans in Retired Asbestos-Exposed Workers
Source: Diagnostics (Basel). 2026 Aug 10;16(16):2519. doi: 10.3390/diagnostics16162519 (PMC13510779; doi:10.3390/diagnostics16162519)

diagnostics

Supplementary Figure S1. Schematic representation of the MIL architecture employed in our workflow. The ResNet-18 backbone processes instance-level features to generate a patient-level prediction of PP presence.

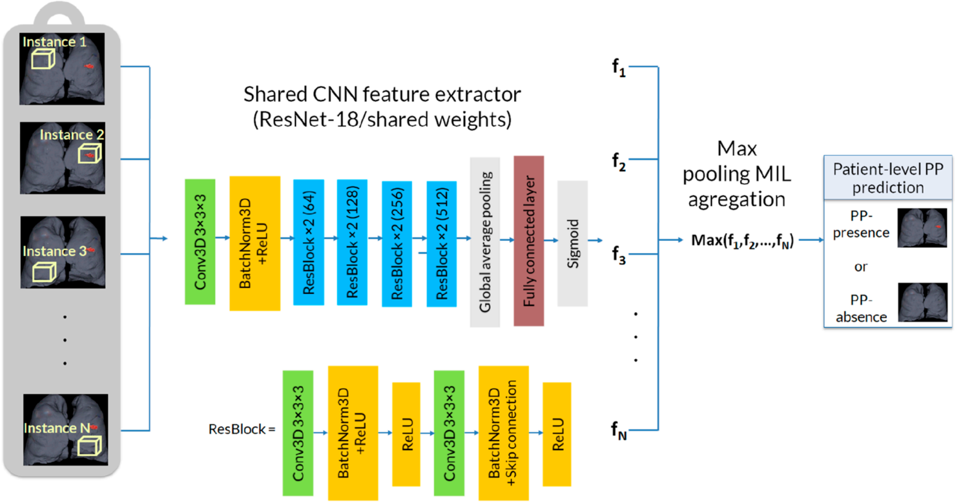

Supplementary Figure S2. Training and validation loss and balanced accuracy curves over 100 training epochs for the ResNet-18 classifier using 9 × 9 × 9 patches generated with the SC-proba strategy (top N = 100 connected components retained). Solid lines represent the mean performance across 10 independent training runs, and the shaded regions indicate the corresponding standard deviation. (a) Training (blue), validation (red), and validation with early stopping (green) loss curves. (b) Training (blue), validation (red), and validation with early stopping (green) balanced accuracy curves. Early stopping was applied based on validation balanced accuracy to mitigate overfitting, and the model with the highest validation balanced accuracy was retained for subsequent evaluation. The green curves illustrate the performance of the selected models after early stopping, confirming its effectiveness in stabilizing validation loss and maintaining high balanced accuracy.

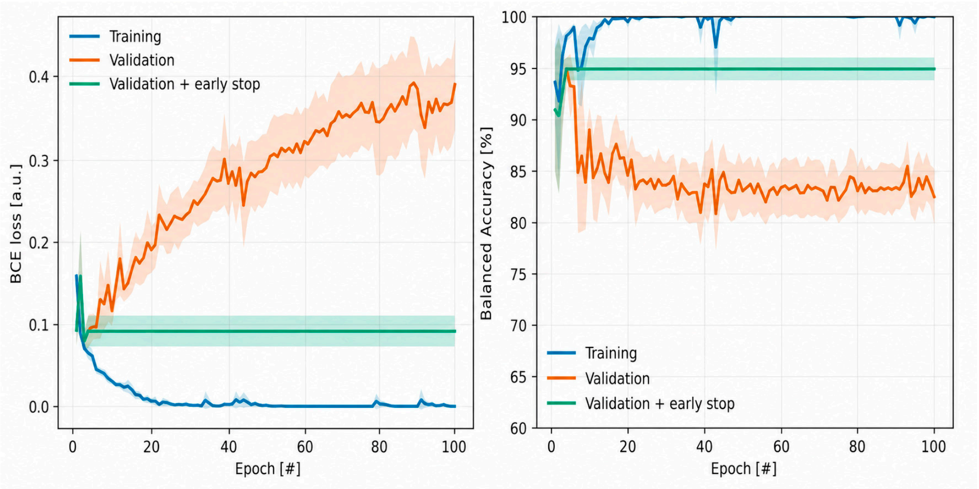

 https://doi.org/10.3390/diagnostics16162519

Supplementary Figure S3. Confusion matrix for patient-level PP detection on the test dataset using the proposed MIL framework. Values represent the mean row-normalized percentages of true negatives (TN), false positives (FP), false negatives (FN), and true positives (TP) across 10 Monte Carlo cross-validation runs.

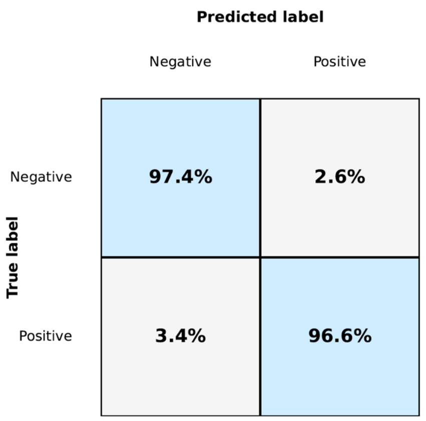

Supplement: Supplementary file 1 [file diagnostics-16-02519-s001.zip › diagnostics-4387300-supplementary.pdf]
